# Reinventing postgraduate training in the plant sciences: T‐training defined through modularity, customization, and distributed mentorship

**DOI:** 10.1002/pld3.95

**Published:** 2018-11-20

**Authors:** Natalie A. Henkhaus, Crispin B. Taylor, Vanessa R. Greenlee, Delanie B. Sickler, David B. Stern

**Affiliations:** ^1^ American Society of Plant Biologists Rockville Maryland; ^2^ Boyce Thompson Institute Ithaca New York; ^3^ Present address: Cornell University Ithaca New York

**Keywords:** career training, evolution/evolutionary genetics, metabolism, plants, science communication

## Abstract

The Plant Science Research Network (PSRN) comprises scientific societies and organizations with a mission to build and communicate a consensus vision of the future of plant science research, education, and training. This report enumerates a set of far‐reaching recommendations for postgraduate training that emerged from workshops held in October 2016 and September 2017. These recommendations broaden and deepen the T‐training concept presented in the Decadal Vision for Plant Science, which emphasizes experiential learning beyond the traditional disciplinary focus. Both workshops used the scenarios developed in Imagining Science in 2035 as a mechanism to encourage out‐of‐the‐box thinking, an approach that led to the innovative recommendations and solutions described here. At the heart of our recommendations is the empowerment of trainees, who should be enabled to customize and take ownership of their training experiences. This fundamental concept is embodied in five principles: (a) Trainees should be provided guidance and resources needed to define and pursue career objectives within and beyond academia, conferring to them greater independence and responsibility in shaping their own future. (b) Learning should be flexible, adaptable, and distributed. Training should combine traditional and modular coursework to encompass both technical and professional skills. Guidance from diverse mentoring teams will support and tailor training toward diverse, personalized career paths. (c) Scientific research experiences should be broad and question‐driven, whether motivated by basic discovery or seeking solutions to societal challenges. Trainees should continue to gain mastery of one or a few core scientific disciplines and their key tools and approaches. (d) Trainees should be skilled in science communication and incentivized to engage with and learn from the broader public community, helping to maintain an active dialogue among public, private, and academic sectors. (e) Training programs should foster and facilitate the inclusion of individuals with a diverse range of life experiences and should prioritize trainee well‐being. The report recommendations call for a profound cultural shift, one that embraces and extends educational delivery trends toward self‐learning and distance learning, considers trainee well‐being as an essential requirement for success, and acknowledges the importance of effective two‐way communication with the public. This shift is intended to broaden participation in the plant science workforce, both in terms of diversity and numbers, while maintaining excellence in core scientific training. Cultural change takes time, but among academic institutions the need for significant change and innovation in postgraduate training is increasingly pressing. As such, the immediate intent is for these recommendations to catalyze pilot programs and also build on emergent prototypes that exist globally while creating momentum for larger scale changes over longer time periods.

## 1 INTRODUCTION

**Figure 1:**
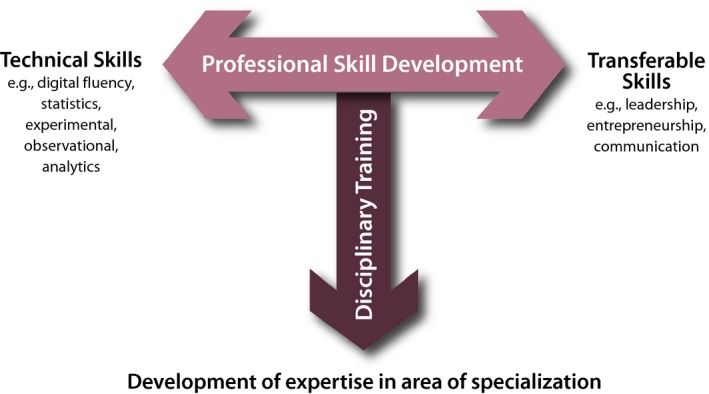
T‐training approach for diverse careers. The T‐shaped individual develops professional skills and takes part in deep disciplinary training. Skills may vary among disciplines but include transferable as well as technical skillsets. Disciplinary training may be acquired through specific research experiences or degree programs. Modularity, customization, and distributed mentorship further support T‐training. Figure adapted from the Decadal Vision report (*Unleashing a Decade of Innovation in Plant Science: A Vision for 2015–2025*, 2013)

The Plant Science Research Network (PSRN) comprises scientific societies and organizations with a mission to build and communicate a consensus vision of the future of plant science research, education, and training. This report enumerates a set of far‐reaching recommendations for postgraduate training that emerged from workshops held in October 2016 and September 2017. These recommendations broaden and deepen the “T‐training” concept presented in the *Decadal Vision for Plant Science*, which emphasizes experiential learning beyond the traditional disciplinary focus (Figure 1) (Unleashing a Decade of Innovation in Plant Science: A Vision for 2015–2025, 2013). Both workshops used the scenarios developed in *Imagining Science in 2035* as a mechanism to encourage out‐of‐the‐box thinking, an approach that led to the innovative recommendations and solutions described.

### 1.1 Core principles

**Figure 2:**
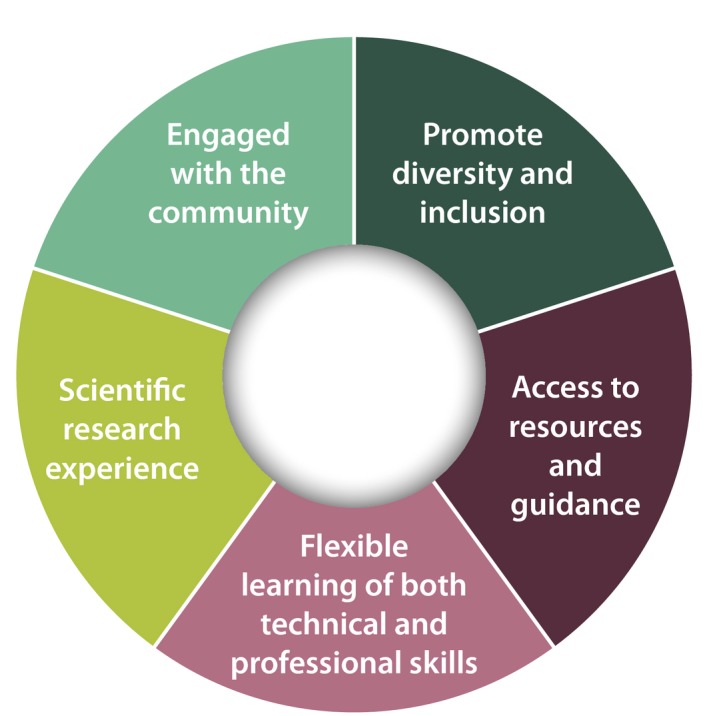
Empowering trainees. Empowering trainees by providing them greater independence and responsibility in shaping their own futures. The five core principles are: prioritization of and support for diversity, inclusion, and trainee well‐being; guidance and resources to define and pursue career objectives; flexible learning, unconstrained by institutional boundaries; disciplinary mastery achieved through research experiences; and community engagement through science communication

At the heart of our recommendations is the empowerment of trainees, who should be enabled to customize and take ownership of their training experiences (Figure 2). This fundamental concept is embodied in five principles:

1. Trainees should be provided guidance and resources needed to define and pursue career objectives within and beyond academia, conferring to them greater independence and responsibility in shaping their own future.
2. Learning should be flexible, adaptable, and distributed. Training should combine traditional and modular coursework to encompass both technical and professional skills. Guidance from diverse mentoring teams will support and tailor training toward diverse, personalized career paths.
3. Scientific research experiences should be broad and question‐driven, whether motivated by basic discovery or seeking solutions to societal challenges. Trainees should continue to gain mastery of one or a few core scientific disciplines and their key tools and approaches.
4. Trainees should be skilled in science communication and incentivized to engage with and learn from the broader public community, helping to maintain an active dialogue among public, private, and academic sectors.
5. Training programs should foster and facilitate the inclusion of individuals with a diverse range of life experiences and should prioritize trainee well‐being.

### 1.2 Specific recommendations

**Figure 3:**
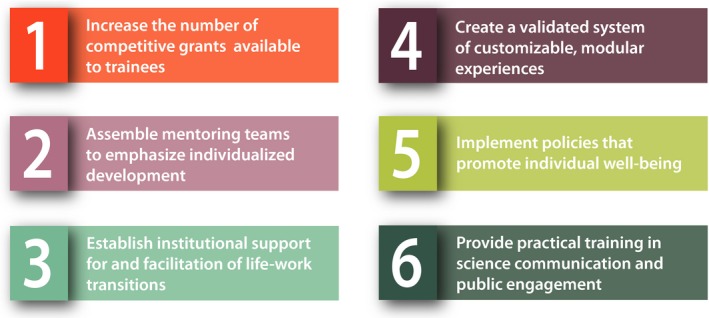
Specific recommendations. The six recommendations to reinvent postgraduate training are related to funding, mentoring, modular training career flexibility, well‐being, and engagement with the broader community

Our recommendations are scalable and can be adapted to various training environments; they also learn from and may be applied to other disciplines (Figure 3).

1. Increase the number of competitive grants available to trainees. Emphasize direct funding of trainees including “gap year” students, graduate students, postdoctoral fellows, and those engaging in continuing education. As the possessors of their own funding, trainees will experience greater ownership of their path, increased choice and mobility, and heightened accountability for their own progress.
2. Rethink mentoring to emphasize individualized development. Encourage the formation of distributed mentoring teams that assemble advisers from job sectors that reflect and support a trainee's personalized aspirations and areas of focus. Individual development plans are recommended to formalize expectations, encourage introspection, foster accountability, and monitor goal‐setting and achievements.
3. Create a validated system of customizable, modular experiences. Develop a modular approach to supplement institutional offerings, comprising e‐learning, short courses, workshops, and internships, thus creating a curriculum that spans institutions, learning methods, technical and professional skills, and topic areas beyond the plant sciences. In parallel, implement a credentialing system that documents and validates learning experiences and acquired skills in a widely accepted format.
4. Establish institutional support for and facilitation of life‐work transitions. Promote opportunities for career flexibility, allowing and encouraging trainees to transition more smoothly from the traditional academic pipeline, so that they may accommodate diverse personal, community, and financial circumstances, and facilitate training throughout the duration of scientific careers.
5. Develop policies to promote individual well‐being. Increase diversity and inclusion through policies that support work‐life balance, mental health, wellness, and family leave.
6. Provide opportunities and practical training to develop communication skills and foster a research environment that promotes two‐way public engagement.

### 1.3 Intended outcomes

These recommendations call for a profound cultural shift—one that embraces and extends educational delivery trends toward self‐learning and distance learning, considers trainee well‐being as an essential requirement for success, and acknowledges the importance of effective two‐way communication with the public. This shift is intended to broaden participation in the plant science workforce, both in terms of diversity and numbers, while maintaining excellence in core scientific training. Cultural change takes time, but among academic institutions the need for significant change and innovation in postgraduate training is increasingly pressing. As such, the immediate intent is for these recommendations to catalyze pilot programs and also build on emergent prototypes that exist globally while creating momentum for larger scale changes over longer time periods.

## 2 BACKGROUND

### 2.1 Advancing the training goals of the *Decadal Vision*

In 2013, the plant science research community published *Unleashing a Decade of Innovation in Plant Science: A Vision for 2015–2025* (the *Decadal Vision;* (Unleashing a Decade of Innovation in Plant Science: A Vision for 2015–2025, 2013)). Among the five major goals described in the *Decadal Vision* is “Reimagining Graduate Training,” which outlines a “T‐training” model (Figure 1) that retains deep disciplinary training as a major component (the vertical part of the “T”) while incorporating a broader palette of professional skills (the horizontal top of the “T”). Our concept of T‐training for the sciences is analogous to calls to train “T‐shaped professionals” that were developed over the past few decades (Donofrio, Spohrer, & Zadeh, 2009). The T‐training concept is embodied in two programs launched in 2014, the year after the *Decadal Vision* was published: the U.S. National Science Foundation (NSF) National Research Traineeship (NRT) program, which supports transformative graduate training models that serve a range of Science, Technology, Engineering, and Math (STEM) careers, and the NIH Broadening Experiences in Scientific Training (BEST) program, which supports innovative approaches to prepare postgraduates for a range of career options (White, 2018; Meyers et al., 2016). Given the resonance of these promising pilot programs, the PSRN sought ways to bring T‐training into the mainstream, using plant science as a focus for postgraduate program conception, but with the expectation that any novel approaches would both learn from and contribute to a range of disciplines.

The PSRN's strategy was to assemble diverse participants for two successive but independent workshops, in both cases creating an environment that stretched minds and encouraged imaginative rather than incremental thinking. The PSRN first constructed a set of four future scenarios that lay out a spectrum of global contexts in which plant science research and training might be playing out 20 years hence. The scenarios were described in the report *Imagining Science in 2035: Strategies for Maximizing the Value and Impact of Plant Science, and Beyond* (*Science in 2035*), and envision and explore a range of possibilities for the types of science that will be emphasized and the manner in which that science will be resourced (Plant Science Research Network, 2016). In the two workshops, participants asked how T‐training could be implemented in each *Science in 2035* scenario before homing in on specific strategies that would be most effective across all four highly divergent scenarios. Participants in the first workshop were predominantly industry scientists, academic faculty, and senior university administrators, whereas the second workshop was restricted to early career trainees (see Appendices S1 and S2). Despite the very different cohorts, their recommendations overlapped extensively and have been merged in the current report.

### 2.2 Recommendations

**Figure 4:**
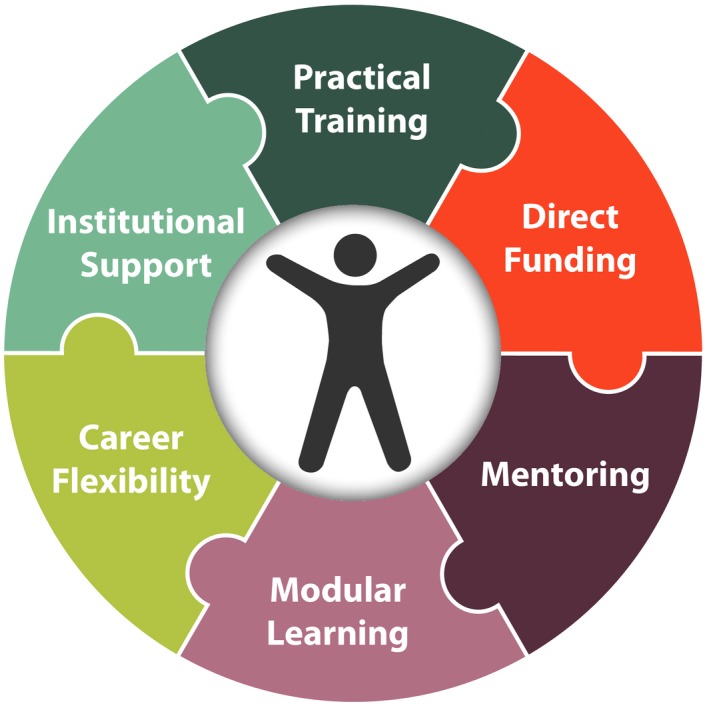
Place the trainee and their needs at the center. Individual development plans (IDPs), which enable mentors and mentees to personalize training, should be used to achieve personal and professional objectives. The trainee‐centric approach comprises six elements: direct funding of trainees; a flexible, multi‐mentor model; modular training that build skill sets outside of degree programs; support for flexible career on‐ramps and off‐ramps; a focus on trainee well‐being; and opportunities for practical training (e.g. science communication, research internships, or experiences in other sectors)

**Figure 5:**
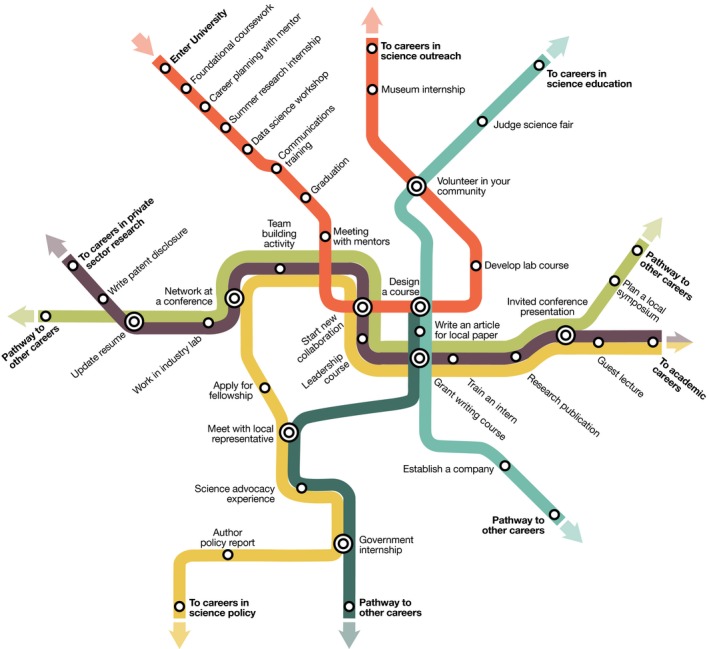
Pathways to diverse careers. An imaginary metro map representing possible career pathways and T‐training opportunities. While some stations are more common entry points, trainees can use any station to enter or exit. Although destinations are neither fixed nor preordained, trainees should be adequately prepared for a range of opportunities and career‐long adaptability. Key: (circles) metro stations represent an activity or development of a particular skillset; (bullseyes) transfer stations represent career transition points; (arrows) each line leads to a different career pathway

In this section we outline six focus areas that will empower trainees to achieve outcomes that align closely with their personal and professional goals (Figure 4). In doing so, we challenged two established perspectives on training, recognizing the simple fact that only a minority of doctoral trainees desire or obtain careers as academic faculty. The first paradigm we challenge is that degree attainment marks the acquisition of a suitable set of competencies (Commission on Creating the Next in Education, 2018; White, 2018; Lohr, 2018). Instead, we believe that success is achieved when the acquired competencies match both the trainee's need to prepare for their preferred career trajectory and the employer's expectations with respect to that individual's competencies and potential. Second, we challenge the pervasive use of “pipeline” terminology, with its impermeable, linear connotation and its susceptibility to imagery of blockages and leaks. Instead, we contextualize training as a network of paths, which may be combined and sequenced throughout one's training and subsequent career to promote preparation for a variety of professional destinations (Figure 5). The specific recommendations for postgraduate training that resulted from the PSRN workshops are described below (Figure 3).

#### 2.2.1 Recommendation 1: Increase the number of competitive grants available to trainees

We recommend increasing direct funding, not only for doctoral students and postdocs, but also for nontraditional students, such as those in gap years and those seeking training to enable workforce re‐entry or career switches. Therefore, although funding would include traditional support, such as NSF or USDA Graduate Research and Postdoctoral Fellowships or NIH National Research Service Awards (NRSAs), we recommend creating new types of short‐ and medium‐term fellowships for specific purposes. The benefits of funding trainees directly include

1. Training driven by intellectual interests, career goals, and individual values, rather than by funding available in a specific laboratory or program;
2. Increased personal ownership of training, reflected in increased enthusiasm and improved engagement;
3. Experience in developing and managing a budget, networking, and technical writing;
4. Increased power to choose the best mentoring environment, incentivizing improvements in mentorship;
5. Increased flexibility and institutional support for trainees to independently pursue external internships and earn credits through online programs, short courses, and workshops offered by entities beyond the research institution;
6. Stimulating universities and other research‐ and training‐focused organizations to develop creative programming and compete for trainee interest.

A direct funding model challenges current practices that match trainees with open research slots, and it will impact the manner in which laboratories are populated. We recommend adequate additional funding that would be awarded directly to trainees, while acknowledging that continued awarding of research grants to laboratories will be required to maintain infrastructure, technical support, and materials.

A second challenge is that trainees will generally benefit from or require sustained attention to ensure that they effectively design and manage their professional development and technical training. Recommendation 2 addresses the need to couple program and mentor accountability with training to ensure that these expanded opportunities fully benefit the trainee.

The need to support lifelong training reflects the fact that career adaptability and mobility can be critical assets, but scientists may be unsure how to learn a new technology or discipline, transition between the public and private sectors, or reenter the workforce after a hiatus. We therefore recommend creating funding programs for professionals and faculty at all types of higher‐learning institutions, as well as for individuals outside academic settings who otherwise may have no recourse to funding to support their continued education and professional development.

#### 2.2.2 Recommendation 2: Rethink mentoring to emphasize individualized development

Giving trainees more autonomy and responsibility challenges current training models. Most graduate students are currently guided predominantly by a single mentor/supervisor, with intermittent advice from a committee that is typically composed of academics at the same institution, who tend to be deferential to the major adviser. As a result, training may occur in an environment that fails to build awareness of, and confer access to, a full range of learning and career options. We recommend an alternative to this model, where trainees develop distributed mentoring teams that have complementary expertise and that are selected to help develop and manage professional goals, which often evolve during training.

Mentoring teams, which could advise both thesis students and nontraditional learners, might be drawn from both active and retired scientists from all sectors of the workforce, including academia, industry, nongovernmental organizations, or public service (Schmidt, 2006). Even if a mentoring team were strictly academic, it should be the rule rather than the exception to include faculty from different fields, as well as from other institutions. New models for committee management should be explored, such as a co‐chair structure that splits primary responsibility between research advising and career counseling. Those mentors whose role emphasizes career counseling should be offered specialized training so that they can provide effective guidance on transferable skill development and career options, and they should be recognized and rewarded for their contributions in this regard.

Access to mentors is envisioned to occur via a network or marketplace. The American Society of Plant Biologists, with its Plantae networking platform, or the National Research Mentoring Network could provide matchmaking resources through which mentors and mentees might connect. This might happen online initially with follow‐up, in‐person connections via “speed dating” or other professional networking events hosted at large conferences or regionally in a specific physical location or city, or within specific subdisciplines of plant science. Such organizations already enjoy active participation by industry and government scientists, so they are primed for trainees who seek to connect with mentors from various sectors and institutions. It is essential that committed mentors are incentivized and rewarded for participating in this marketplace, which goodwill alone might be insufficient to populate. Trainees should also receive guidance on how to make the connections so that they can identify appropriate mentors and engage with them in long‐term, mutually rewarding professional relationships.

To ensure that mentoring experiences are effective and expectations are clearly enumerated, we recommend that trainees create and maintain Individual Development Plans (IDPs) that establish training and career goals and, importantly, set out realistic pathways toward achieving them (see Figure 5) (Austin & Alberts, 2012; Hobin, Fuhrmann, Lindstaedt, & Clifford, 2012). The trainee's IDP would be implemented for the duration of a training period and might incorporate coursework, internships, field experiences, learning modules, and major experimental themes and timelines. The IDP would provide a framework for identifying skills that coincide with potential career interests of the trainee and allow the trainee to build those skills in a manner that is efficient, effective, and fulfilling.

Accountability is an important facet of a strong mentoring system. The plan holds the trainee accountable, with funding potentially being contingent on creating and updating the IDP. Mentors also need to be held accountable, perhaps through oversight of a networking platform, which could incorporate both a rating system and an annual record of the type and frequency of meetings held. Annual reports are common practice to monitor the research progress of graduate trainees, but professional career development is not often a component of those reports. Institutional oversight will also need to be incorporated into a revised mentoring model to select and monitor the source of external mentors and to help ensure that their contributions are recognized.

#### 2.2.3 Recommendation 3: Create a validated system of customizable, modular experiences

Acquiring the skills laid out in IDPs will require a more diverse assortment of learning experiences than those prevalent in today's typical postgraduate training environments. Thus, traditional laboratory or classroom experiences will need to be supplemented by alternative sources and delivery mechanisms. We recommend the creation of a modular system that includes e‐learning, short courses, workshops, and internships (Massive Open Online Courses Help Make STEM Education More Accessible, But Do They Work for All Students?, 2013; Monetization over Massiveness: A Review of MOOC Stats and Trends in 2016 — Class Central, 2016). The result will be a flexible, customizable curriculum that spans disciplines, institutions, learning methods, career stages, and career options. A widely recognized and validated credentialing system, developed in collaboration with employers, will be required to document skill acquisition.

Possibilities abound for topics and forums to develop into learning modules. The addition of training modules for broader learning aims will amplify, rather than impinge upon, the core scientific research experience. Modules may help to reinforce the nature and excitement of discovery while imparting indispensable skills such as hypothesis development and testing, project management, and data analysis. Empowerment and customization, therefore, must be blended into a system in which research and analytical progress are still emphasized.

How will the academic, corporate, government, and other employer communities evaluate and credit completion of modules? Without a formal system in place, there is risk that those accomplishments may be regarded with skepticism or uncertainty by potential employers. Time to the Ph.D. degree may lengthen beyond its already unpalatable duration, especially if the trainee's home institution fails to credit such training. Current practices of accepting Advanced Placement credit, along with limited amounts of transfer credits or summer coursework from other accredited institutions, demonstrate the potential of this approach. Although management of “alternative credentials” is still in its infancy, it is clearly on the radar of higher education (Buban, 2017).

We recommend that access to, and credentialing of, modular training for plant science be managed jointly. Access could be optimized by establishing a “one‐stop” repository for accredited learning opportunities, with a suitable organizational framework for searches. At present, there is no widely accepted gateway, leaving trainees to rely on career offices, mentors, word of mouth, and web searches. One possibility for content management would be to develop a consortium of professional societies, content providers, and academic representatives. This is not a trivial exercise, but shifting toward a common platform that reflects buy‐in from PSRN members, professional societies, universities, and industry would help to standardize and organize resources and would expose students more broadly to plant science and the range of career trajectories available to them (see Pilot 8 in Appendix S3).

#### 2.2.4 Recommendation 4: Establish institutional support and acceptance for work–life transitions

Currently, leaving for employment without completing a degree tends to be regarded as making the best of a failure; our recommendation is to regard the exit as a positive, strategic choice, so long as it is deliberate and has been planned for. We recommend a wider acceptance of, and preparation for, career transitions that do not coincide with completion of a degree and may not even envision a degree from the beginning. Such cafeteria‐style curricula are currently under consideration within academia (Commission on Creating the Next in Education, 2018). Also, the credentialing system outlined above could be leveraged to validate an individual's competencies and skills beyond or outside of an academic curriculum.

Degree‐independent training would provide support for work–life transitions that currently might derail a career. Economic, family, or other considerations might call for one or multiple transitions that could be buffered by retraining that had the benefit of a visible structure of learning modules, mentoring, and possible financial aid. It is additionally likely that this type of framework would assist in broadening participation among groups currently underrepresented in science, who disproportionately face economic or institutional barriers.

The specter of economic barriers is also raised by the 2018 Science & Engineering Indicators published by the National Science Board (National Science Board, 2018). As one example, the time to Ph.D. degree is 7.3 years in agricultural fields overall, but 8.7 and 8.5 years for Hispanic and African American students, respectively. Furthermore, it is debatable whether receiving a doctoral degree will translate into greater earning power in the years ahead. Individuals with biology Ph.D.'s awarded between 2009 and 2011 were found to earn $36,000 in the year after their doctorate, or in the mid‐$40,000 range if postdoctoral appointments are excluded (Zolas et al., 2015). Longer‐term prospects are more favorable, but one must account for the seven or more years of minimal income and the effects on retirement savings (Bureau of Labor Statistics, 2017). Graduate student stipends are scarcely sustainable for many trainees, especially those with family responsibilities or undergraduate student loan debt. These facts underscore that alternatives to formal degree paths are urgently required.

#### 2.2.5 Recommendation 5: Develop policies to promote individual well‐being

It is well documented that diverse teams are more effective and make better decisions when the participation of all team members is encouraged and equally valued. Moreover, there is a widely held imperative to work actively toward enabling a STEM workforce that mirrors the demographics of the broader population. Although the present report does not make specific recommendations as to how to broaden participation in the plant sciences, we believe that several of the recommendations will help lower barriers to attracting and retaining diverse populations. These include IDPs and mentoring, which set out goals that are attainable and with purpose, along with funding that supports a variety of career pathways (Roach & Sauermann, 2017). Additionally, modular professional development offerings allow trainees to align career‐building activities with other responsibilities in an individualized manner. Third, flexible career transitions allow commitments to be scaled to the realities of an individual's life. Finally, there are relevant communications skills, which are set out in the next section of this report.

Discussions during the PSRN training workshops often returned to the issues of work–life balance and trainee well‐being. It is clear from these discussions and from recent research that the current expectations of trainees in most academic research labs frequently run counter to these principles and can directly contribute to depression and other mental health disorders (Evans, Bira, Gastelum, Weiss, & Vanderford, 2018). Furthermore, these expectations, especially when coupled with the long‐term commitments required to pursue graduate degrees and many postdoctoral appointments, will increase retention among all trainees and help nurture a diverse workforce. We contend that scientific excellence and advancement are not and should not be incompatible with leading a balanced life and that improved work–life balance will lead to better research and professional outcomes. We therefore recommend broad adoption of programs and policies that support individual well‐being and work–life balance, including mental health and wellness, and family leave.

#### 2.2.6 Recommendation 6: Provide training in science communication

Workshop participants defined two major categories of communication skills: those internal to a research career and those that connect and engage scientists with the broader non‐research community. Training and development in both categories are essential to achieve an adequately prepared future plant science workforce.

Individuals pursuing research‐intensive careers deploy a range of communication techniques aimed at colleagues, including creating and delivering poster or slide presentations, technical writing, and teaching. Most graduate programs have formal or semiformal ways of delivering this training. We recommend adding richness to this training in two ways. First, ethics training should be compulsory. While scientific misconduct is more frequently covered, the gray areas prevalent in collaborative work, including unconscious bias, are ripe for exploration and will enable researchers to handle issues related to credit, responsibility, and scientific disagreements with more confidence. Collaborations increasingly involve biologists of many stripes, as well as data scientists or engineers, and the capacity to communicate effectively across these boundaries is critical but often does not come naturally (Friesner et al., 2017; Plant Science Research Network, 2017). Furthermore, trainees equipped with such skills will tend to be more effective in team‐oriented workplaces, particularly in the private sector.

In terms of engendering two‐way communication among scientists and the broader communities such as journalism, public policy, and the general public, the PSRN recommends the development of outreach programs that enhance dialogue among scientists and their local communities. This might include schools, local news organizations, and government agencies. Specific communication skills are required to build trust between scientists and the public. Among them are the use of appropriate vocabulary, the capacity to establish empathy, framing the message, and being an attentive listener. In general, scientists are taught to project a message and answer scholarly questions, but not taught listening skills for other audiences and how to be appropriately responsive. Scientists have not been particularly successful at depolarizing topics such as genetic modification, climate change, or vaccine safety. While the training of scientists would not be a one‐stop solution, it is an essential component.

The proposed modular system is ideal for developing diverse communications skills. Collaborative skill development could occur on campus—for example, in courses shared with communications, journalism, computational, or engineering departments, or through other campus affiliates (see Pilot 6). Learning from peers lowers barriers and builds a sense of community, and these topics are amenable to online formats, where they can be widely disseminated. We also recommend offering focused workshops or courses to provide trainees with experiential communication opportunities; these might be made available on campus or during scientific meetings and could be sponsored by individual laboratories, professional societies, companies, or educational institutions.

Reaching into communities will require a more direct approach and will likely have a larger impact if it is initiated by scientists from the same geographic area, and especially when there are ethnic or socioeconomic commonalities (see Pilot 5). Alignment with programs targeted to improve K–12 and undergraduate education in plant science would build efficiency and also bring forward opportunities for citizen science. On‐campus student associations or clubs could be encouraged to develop outreach programs and be incentivized through a reward system. Such activities are fully compatible with the Land Grant mission, where plant science is a major component.

## 3 THE CASE OF THE POSTDOC

### 3.1 How PSRN recommendations might impact post‐Ph.D. training and trajectories

Most of the recommendations above apply equally to graduate students and postdoctoral scientists, all of whom would benefit from garnering independent funding, use of IDPs, access to modular training, and improved communication skills. The question that is not addressed, however, is the proper role of postdoctoral training over the coming decades. To wit, none of the four scenarios that make up *Imagining Science in 2035* played out in our workshops featured postdoctoral training; in other words, the hypothetical trainee, Dakota, did not seek or require postdoctoral training to achieve career success.

At present, postdoctoral training is commonly sought by life sciences Ph.D. holders to gain specialized skills or to work with a specific scientist. Such training can be invaluable and is generally considered to be a prerequisite for both industry research team leader and faculty positions in the life sciences, although such a requirement varies across fields (‘Has the Use of Postdocs Changed?’, 1998). A significant number of today's trainees report, however, that they entered postdoctoral study primarily as a cultural expectation or because they were unable to secure other employment (National Science Foundation, 2015). Not infrequently, these experiences turn into “permadocs,” that is, lengthy appointments with diminishing chances of career advancement into independent positions that are buffeted by adverse impacts on family life, ranging from inconvenience to extreme stress (Academics anonymous Universities, 2016; Beryl Lieff Benderly, 2015; Hendrix, 2017; Powell, 2015).

Dismay with such outcomes could be contributing to the decline in numbers of biology postdocs, which has been partly balanced by an increase in “nonfaculty researchers” as discussed below (Arbeit & Kang, 2017). Postdoc distress may also contribute to poor perceptions of career opportunities among more junior researchers, who instead opt for seemingly less arduous or more lucrative trajectories. As the recommendations described above take hold, however, it is reasonable to expect that an increasing proportion of postgraduate trainees will reinforce this trend by identifying their preferred career trajectories much earlier and receiving the training, experiences, and mentoring they need to achieve their objectives.

Insofar as a time‐limited postdoctoral training period remains a logical and legitimate requirement for certain career objectives, we support implementing this report's recommendations. Although the NSF already requires a mentoring plan for postdocs supported on grants that includes professional development and career counseling, this principle should become universally applied and much more effectively organized. Where appropriate, this plan could also tap into credentialed modular and external training experiences, as well as mentors, that map to expectations and objectives laid out by postdocs through their IDPs. Considerable expansion of portable postdoctoral fellowships would confer trainees with greater ownership and mobility, and these fellowships would provide incentives for laboratory heads to emphasize “added value” training beyond the research experience, such as richer and deeper experience in teaching, writing, development of scientific research projects, and opportunities for mentoring, serving on committees, peer review, and learning more about laboratory leadership and project management. Suggestions made for NSF postdoctoral mentoring plans are a useful touchstone for conceiving such objectives (GPG Chapter II, 2010).

Whether a stronger alignment between postdoctoral training and specific career trajectories results over time in more or fewer postdocs in any given discipline, a shift in the constitution of the experimental workforce must be contemplated. In some of the *Imagining Science in 2035* scenarios, robotics takes on a major role as many of the more repetitive tasks in laboratories are automated. However, we do not envision the demise of the human scientist. An emerging alternative to permadocs is nonfaculty researcher positions, which NSF defines as individuals involved principally in research activities who are not postdocs or members of university faculties. These positions might include long‐term research staff, technicians, system administrators, collaborative team leaders, community managers, and laboratory managers, among others.

### 3.2 Pathway to implementation

The PSRN training recommendations call for a cultural shift over a 20‐year time frame toward empowering trainees to develop and complete customized training pathways (Figures 4 and 5). Recognizing this, the PSRN envisions two main implementation phases. Phase 1 would involve the piloting of new support mechanisms (e.g. mentoring teams and associated IDPs) at multiple locations. This support would be supplemented with evaluation of existing relevant pilots (such as the previously mentioned NRTs, the BEST program, and the Foundation for Food and Agriculture Research's recently announced graduate fellowship program, https://foundationfar.org/ffar-fellows/). It would also involve the documentation and development of modular training experiences and mechanisms for credentialing them. Phase 2 would incentivize the expansion of successful pilots from Phase 1 (see Appendix S3).

### 3.3 Phase 1: Developing pilot programs

Pilot program concepts have been developed both during the writing of this report and as a component of the September 2017 PSRN workshop. In the latter case, the major contributors are credited. The complete programs are fully described in Appendix S3 and summarized here. These ideas are intended either for implementation or to stimulate the creation of additional pilot concepts.

#### 3.3.1 Pilot 1: Unconventional training through direct funding

Direct funding is critical to encourage nontraditional entry into science training pathways. This pilot would make awards to support career‐switching, workforce re‐entry, retraining, or nondegree training to fill out a resumé. Existing laboratories accommodating such trainees would also help to fulfill the broader impacts aspect of NSF‐supported research.

#### 3.3.2 Pilot 2: Team mentoring

*Element 1 ‐ Mentor databases*. Resources populated with volunteers from academia, industry, and elsewhere could be built to centralize and democratize access to advising.

*Element 2 ‐ Alternative mentoring team structures*. Graduate mentoring teams with multiple leadership roles, as distinct from the single chair structure, could be piloted.

*Element 3 ‐ Providing support resources*. Roles and responsibilities on the mentoring team, and even roles among the broader mentoring community, could be defined and clarified, along with recommended best practices for guiding a trainee through the development of an IDP.

#### 3.3.3 Pilot 3: Building a successful mentoring team

Contributors: Shandrea Stallworth, Valerie Fraser, Natalie Henkhaus, Katie Murphy, Andre Naranjo

This pilot would assist undergraduate and graduate students, postdocs, and young faculty members in developing meaningful and useful mentor/mentee relationships and teams in academia and industry.

#### 3.3.4 Pilot 4: Developing and credentialing training modules

*Element 1 ‐ Complementary experience grants*. Funding agencies could offer short‐term fellowships for students to undertake research or learning activities distinct from their main research project.

*Element 2 ‐ Credentialing*. Credentialing models for modular learning could be piloted, or existing ones could be enhanced or optimized for plant science.

*Element 3 ‐ Warehousing*. A repository of accredited modules goes hand in hand with credentialing. Therefore, plant (and life) science will require its own databases housing the fairly eclectic collection of opportunities that trainees may be seeking.

#### 3.3.5 Pilot 5: Industry and academia conference for students

Contributors: Emma Frawley

The development of a 2‐ to 3‐day conference with two main objectives is proposed: first, to foster an environment to understand and improve the relationship between academia and industry, and second, to facilitate trainee networking with plant science–related industries.

#### 3.3.6 Pilot 6: Science communication training

Contributors: Nicole Forrester, Nat Graham, Chris Barbey

Communication skills are essential for successful careers in science, yet students and researchers have limited opportunities to acquire these skills during their academic training. To address this gap in training, a series of free videos focused on communication skills is proposed that can lead to a credential.

#### 3.3.7 Pilot 7: Diversity workshop to increase participation of underrepresented groups in the plant sciences

Contributors: Andrea Carter, Chelsea Pretz, Ashleigh Farmer, Nathan Vega

The purpose of this workshop is to bring together representatives from industry and the academic community—to include students and administrators involved in student diversity programming—to discuss how to increase involvement of underrepresented groups in plant science. The ideas presented in the pilot were forerunners to a PSRN‐HHMI workshop on broadening participation to be held in January 2019.

#### 3.3.8 Pilot 8: MAYS: Navigating and networking your career in plant science

Contributors: Megan Kelly, Megan Sylvia, Crispin Taylor

A multimedia approach is proposed to address lack of readily available information regarding career pathways in plant science.

#### 3.3.9 Pilot 9: Pop‐up leadership academy

Contributors: Hallie Thompson

A pop‐up leadership academy is proposed, which would bring together the concepts of training scientists in nontraditional skill sets via venues that do not rely on classic education through universities or laboratories in a credentialed manner. Focus would be leadership best practices, instilling a culture of continued learning, and developing a sustainable model for training continuation via volunteer curation.

#### 3.3.10 Pilot Program 10: Creating active participants out of trainees

Contributors: Andrew Nelson, Navadeep Boruah, Bethany Huot, Irene Liao

This proposal encourages the funding of regional training hubs consisting of academic, industry, and affiliated plant science groups, which would be the site of transition‐year training programs. Trainees would spend a year sampling different research/affiliated groups and acquiring the transferable skills necessary to take ownership of their future training.

### 3.4 Phase 2: Incentivizing wide adoption of successful pilot programs

Phase 2 has two main components: evaluation of pilot programs and incentivizing the broad adoption of the successful and impactful ones. Thus, pilot programs, when fully developed, should include appropriate metrics as well as the capacity to collect allowable data that will facilitate longitudinal studies. In short, a pilot program should, at its inception, address the question, “what would success look like?” Those that achieve success can serve as models, as a whole or in part.

#### 3.4.1 Evaluation

Short‐term pilot programs suffer from small datasets and a lack of longitudinal information. Simple evaluation mechanisms are appropriate in such cases that match specific inputs (activities) to desired outputs/goals within a framework of the desired long‐term objective. Guidelines are available to select and employ evaluation protocols (Torchim et al., 2012). Where appropriate, pilot program outcomes should also be assessed from the perspective of employers, whether academic or otherwise.

#### 3.4.2 Incentivization

A model of direct funding creates an institutional incentive to optimize training, because prospective trainees, empowered by carrying their own support, will vote with their feet. Furthermore, one trusted source of graduate program rankings is the National Research Council (NRC), whose criteria include several that are directly linked to the proposals put forth here. For example, percentage of first‐year students with external funding, proportion of interdisciplinary faculty, various measures of diversity, and number of student support activities all play into NRC rankings (Carney, Martinez, Dreier, Neishi, & Parsad, 2011). By attracting diverse first‐year students with fellowships and providing a rich training environment, a program would be likely to improve its ranking. Not only may rankings matter to prospective students, but they often matter a great deal to upper administration and donors.

Incentives can also come from the funding side. For example, NRT awards come with certain requirements for the host institution, and in some cases, such as GAANN awards from the Department of Education, cost sharing is also required. For individual rather than site awards, there can also be incentives for institutional commitment. For example, NSF BIO Postdoctoral Fellows are required to have a sponsoring scientist statement that shows “how the proposed host(s) and host institution(s) provide the best environment for the Fellow's proposed research and training plan.” Similarly, USDA‐NIFA pre‐ and postdoctoral fellowships require “productive and interactive mentoring” and “appropriate and applicable training activities.” Institutions and trainers will respond as such criteria are worked into peer review of their applications.

## 4 CLOSING REMARKS

Academia is being challenged to change to keep pace with national needs that include preparing a workforce made up of individuals who are adaptable, quick learners, and adept at communicating across boundaries. Digital fluency is an absolute requirement: five of the largest six U.S. companies are in the technology space, with traditional manufacturing and services lagging behind (Kiesnoski, 2017). At the same time, however, our universities will be drawing their clients from a well of increasing socioeconomic diversity, suggesting that the need to balance family and career obligations will expand (The Changing Face of U.S. Higher Education, 2016).

The personalization and modularization of training articulated in our recommendations resonate with everyday experiences driven by social media, relentless improvements in data analysis and targeting, the needs of the private sector, and the ability to customize most of the interactive world around us. Whether such a world is desirable or not is largely beside the point; science will either learn to function within it, or it will lose its societal support and find itself adrift. This is an end that will serve no one and militates for bold actions that the plant science community is poised to lead.

## AUTHOR CONTRIBUTIONS

Crispin Taylor: study concept and design, drafting of manuscript. Natalie Henkhaus: study concept and design, drafting of manuscript, manuscript assembly, figure design. Vanessa Greenlee: study concept and design, drafting of manuscript. Delanie Sickler: drafting of manuscript. David Stern: study concept and design, drafting of manuscript, figure design. Plant Science Research Network: workshop attendees contributed to the concepts presented in this manuscript.
