# Supplementary material for: Reinventing postgraduate training in the plant sciences: T‐training defined through modularity, customization, and distributed mentorship
Source: Plant Direct. 2018 Nov 20;2(11):e00095. doi: 10.1002/pld3.95 (PMC6508785; doi:10.1002/pld3.95)
Supplement: Supplementary file 1 [file PLD3-2-e00095-s001.docx]

# Appendix 1. Workshop Attendees

**Workshop 1: October 18–20, 2016, in Rockville, MD**

*Participants*

Sally Assmann, Pennsylvania State University, University Park, PA

David Baltensperger, Texas A&M University, College Station, TX

David Baum, University of Wisconsin–Madison, WI

Paul Chomet, NRGene Ltd., St. Louis, MO

Philip Clifford, University of Illinois at Chicago, Chicago, IL

Michael Donoghue, Yale University, New Haven, CT

Vanessa Greenlee, Boyce Thompson Institute, Ithaca, NY

Natalie Henkhaus, American Society of Plant Biologists, Rockville, MD

Toni Kutchan, Donald Danforth Plant Science Center, St Louis, MO

Beronda Montgomery, Michigan State University, East Lansing, MI

Seth Murray, Texas A&M University, College Station, TX

Barbara Olds, Colorado School of Mines, Golden, CO

Emilio Oyarzabal, Monsanto, Chesterfield, MO

Leland (Sandy) Pierson, Texas A&M University, College Station, TX

Chelsea Specht, University of California, Berkeley, CA

David Stern, Boyce Thompson Institute, Ithaca, NY

Crispin Taylor, American Society of Plant Biologists, Rockville, MD

*Guest Speakers*

Jeremy Berg, *Science*

Jeff Hancock, Stanford University

Jacqueline Heard, CiBO Technologies, Cambridge, MA

Isha Ray, University of California, Berkeley, CA

Adrian Taylor, 4Sing: Foresight and Strategy for Security and Sustainability in Governance

*Facilitator*

Susan Stickley, Stratus, Inc., Philadelphia, PA

**Workshop 2: September 19–21, 2017, in Baltimore, MD**

*Participants*

Chris Barbey, University of Florida, Gainesville, FL

Navadeep Boruah, University of Maryland, College Park, College Park, MD

Fernanda (Maria) Buanafina, Pennsylvania State University, State College, PA

Andrea Carter, University of Arizona, Tucson, AZ

Ashleigh Farmer, Claflin University, Orangeburg, SC

Nicole Forrester, University of Pittsburgh, Pittsburgh, PA

Valerie Fraser, Oregon State University, Corvallis, OR

Emma Frawley, Saint Louis University, St. Louis, MO

Nat Graham, University of Minnesota, Minneapolis, MN

Vanessa Greenlee, Boyce Thompson Institute, Ithaca, NY

Natalie Henkhaus, American Society of Plant Biologists, Rockville, MD

Farzad Hosseinali, Texas A&M University, College Station, TX

Bethany Huot, Michigan State University, Lansing, MI

Megan Kelly, Rutgers University

Irene Liao, Duke University, Durham, NC

Katherine Murphy, University of California, Davis, Davis, CA

Andre Naranjo, University of Florida, Gainesville, FL

Andrew Nelson, University of Arizona, Tucson, AZ

Kyle Palos, University of Arizona, Tucson, AZ

Chelsea Pretz, University of Colorado–Boulder, Boulder, CO

Shandrea Stallworth, Mississippi State University, Starkville, MS

David Stern, Boyce Thompson Institute, Ithaca, NY

Megan Sylvia, Pennsylvania State University, State College, PA

Crispin Taylor, American Society of Plant Biologists, Rockville, MD

Hallie Thompson, University of Missouri, Columbia, MO

Nathan Vega, California State University, Fullerton, Anaheim, CA

Qiguo Yu, Waksman Institute of Microbiology, Rutgers University, Edison, NJ

*Guest Speakers*

Maria Wheeler-Dubas, Phipps Conservatory, Pittsburgh, PA

Sarah D. Evanega, Cornell University, Ithaca, NY

*Facilitator*

Susan Stickley, Stratus, Inc., Philadelphia, PA

# Appendix 2. Workshop Agendas

**Workshop 1: October 18–20, 2016, in Rockville, MD**

Plant Science Research Network Postgraduate Training Strategic Retreat

**Location**

Hilton, Washington DC / Rockville Hotel and Executive Meeting Center

1750 Rockville Pike, Rockville, MD 20852-1699

**Objective**

To identify recommendations for action by the PSRN and broader plant science community to enable the potential of the plant sciences through postgraduate training and cyberinfrastructure.

**Tuesday, October 18, 2016**

12:30 pm Registration PSRN Staff

1:00 pm Kick-off: Welcome and Stage Setting

Opening Remarks David Stern

Table Introduction and Discussion

What do we mean by Plant Science?

Keynote Address

How entrepreneurism may shape the future of plant science research.

Jacqueline Heard (CiBO Technologies, Chief Strategy Officer)

Enabling the Plant Sciences: Training and Cyberinfrastructure Discussion of the overarching synergies between postgraduate training and cyberinfrastructure. What is the key underlying question associated with that synergistic space? Table Discussion

3:15 pm Coffee Break

3:30 pm Scenario Planning and PSRN 2035 Scenarios

Presentation to introduce participants to both the scenario thinking methodology and the PSRN scenarios. Susan Stickley

Early Indicators

Discussion of the early indicators that participants identified associated with the PSRN scenarios.

6:00 pm Keynote Address

Big Questions and Big Data

Jeremy Berg (Associate Senior Vice Chancellor for Science, Editor in Chief, *Science*)

**Wednesday, October 19, 2016**

8:30 am Stage Setting and Morning Reflections David Stern and Susan Stickley

Stretching Our Thinking on the Future of Postgraduate Training

Conversations with three provocateurs on how this challenging and changing world can transform the role and model of postgraduate training.

Provocative Speakers

Adrian Taylor (Consultant, 4Sing: Foresight and Strategy for Security and Sustainability in Governance)

Jeff Hancock (Professor of Communications, Stanford University)

Isha Ray (Professor of Energy and Resources, UC Berkeley)

Panel Moderator

David Stern (Professor, President, Boyce Thompson Institute) Interview Series

10:30 am Break

10:45 am Scenario Deep Dive

Exploration of the strategic implications of the PSRN scenarios on the future of postgraduate training in the plant sciences. Breakout Groups

12:15 pm Lunch

1:00 pm Scenario Deep Dive

Breakout Groups

Sharing Insights

Breakout groups present their discussion highlights and strategic recommendations for action in plant science postgraduate training.

Robust Strategies

Identification of strategies that work well across the full set of PSRN scenarios.

3:15 pm Break

3:30 pm Deeper Exploration of Learnings: Competencies of the Future

Based on our strategic exploration of the PSRN scenarios, what will be the key competencies of scientists? How should this inform strategies in cyberinfrastructure?

Changing Legacy Practices

What needs to be addressed in the plant science community culture and practices to create the future we desire?

Next Steps, Closing Remarks David Stern

5:00 pm Adjourn for Evening

**Thursday, October 20, 2016**

9:00 am Morning Reflections, Prep for Presenting Strategies and Recommendations

Opportunity to review and refine learnings and content to share back.

10:15 am Sharing Our Insights

Groups share recommendations from their postgraduate training and cyberinfrastructure sessions.

Enabling the Plant Sciences: Exploring the Shared Space

What are the critical learnings in the shared space of cyberinfrastructure and postgraduate training that enable plant science research? Which insights can be applied to other sciences?

12:15 pm Working Lunch

Discussion of takeaways for action and the critical next steps to create momentum and progress forward. PSRN’s role in progressing the agenda.

1:00 pm Our Path Forward

Compilation of critical next steps moving forward.

1:45 pm Closing Remarks David Stern

2:00 pm Adjourn

**Workshop 2: September 19–21, 2017, in Baltimore, MD**

Plant Science Research Network Workshop to Address Postgraduate Training in the Plant Sciences

**Location**

The Westin Baltimore Washington Airport – BWI, Salon 2

1110 Old Elkridge Landing Road, Linthicum Heights, MD 21090

**Pre-work assignment**

Thoughtfully read Imagining Science in 2035 (bit.ly/ImaginingScience)

Come with an early indicator for each of the four scenarios.

**Tuesday, September 19, 2017**

6:00 pm Registration

6:45 pm Welcome Remarks

Keynote Speakers and Provocative Conversations

Maria Wheeler-Dubas, Ph.D. (Phipps Conservatory and Botanical Gardens)

Sarah Davidson Evanega, Ph.D. (Cornell University)

Panel Moderator: David Stern (Boyce Thompson Institute)

9:30 pm Adjourn for Evening

**Wednesday, September 20, 2107**

8:30 am Kick-off: Welcome, Introductions, Stage Setting

Opening Remarks

Table Warm-up: What Do We Mean by Plant Science? David Stern

9:15 am Scenario Planning and PSRN 2035 Scenarios Susan Stickley

9:45 am Early Indicators

10:30 am Break

10:45 am Scenario Deep Dive

12:15 pm Lunch

1:00 pm Scenario Deep Dive, cont.

1:45 pm Sharing Insights

2:15 pm Robust Strategies

3:15 pm Break

3:30 pm Deeper Exploration of Learnings: Competencies of the Future

4:20 pm Our Strategic Planning Process and Earlier Findings Changing Legacy Practices

5:00 pm Adjourn for Evening

**Thursday, September 21, 2017**

8:30 am Morning Reflections

9:15 am Comparing the Results What’s Consistent? What’s New? What Assumptions May Be at Play?

10:15 am Break

10:30 am Robust Strategies Taking Forward – Closing on the Final Content

11:45 am Enabling the Plant Sciences: What Have We Learned?

12:15 pm Working Lunch

1:00 pm Our Path Forward

1:45 pm Closing Remarks David Stern

2:00 pm Adjourn

# Appendix 3. Pilot Programs

## Pilot 1: Unconventional Training Through Direct Funding

**Contributors:** Report writing committee

Direct funding is critical to encourage nontraditional entry into science training pathways. For example, it will be important to provide financial support for entering training that is unconventional because it occurs mid-career, or because it is not targeted toward completing a degree program. Support could take the form of short-term internships for workforce reentry (e.g., after caring for a family member, return from military service, a career switch). A program might include annual evaluation of the trainee’s progress and career goals, including a discussion of an IDP with an appropriate program mentor. Flexible training could also allow for part-time work or be used for continuing education in combination with an apprenticeship, later developing into full-time employment.

It is very unlikely that a PI would develop a grant budget with funding for a career-switching trainee, but, on the other hand, if such a person made themselves known with specific objectives and funding in hand, they would often be welcomed. In some respects, this is a parallel to Research Experience for Undergraduates (REU) programs, where undergraduates are awarded stipends and join participating laboratories that might otherwise lack their own resources. Accommodating nontraditional trainees through new programs would also help to fulfill the broader impacts aspect of NSF-supported research.

We view the academic research community as being in transition from one that is more focused on developing future faculty to one in which the traditional academic track becomes one among many other equally legitimate and rewarding career (re)development paths. Acceptance will create a virtuous cycle with trainees, who will, over time, lose their reticence to seek these paths. It is already common for conferences to feature professional development workshops with presentations by scientists who “ended up” in technology transfer, law, venture capital, public service, etc. The next steps are to make these destinations more transparent and to match each person’s training to their intended individual pathway or destination.

## Pilot 2: Team Mentoring

**Contributors:** Report writing committee

*Element 1 - Mentor databases.* Resources populated with volunteers from academia, industry, and elsewhere would be built to centralize and democratize access to advising. Although some mentors might add themselves to these databases out of goodwill or individual interest, establishing additional incentives might also be worthwhile. For example, an institution or company could use such participation as a positive criterion in performance evaluation, and a research funder might view active participation as a positive criterion in proposal reviews and project reports.

*Element 2 - Alternative mentoring team structures*. Graduate mentoring teams with multiple leadership roles, as distinct from the single chair structure, could be piloted. For example, an interdisciplinary program might make an ideal testing ground for distributed responsibility of research mentoring. In more traditional research contexts, poles of responsibility might be set up along mentoring modes, with one cochair tracking research, for example, and a second cochair tracking education and career development. Such activities would allow institutions to recast the dominant model that a given trainee is solely associated with a particular faculty member, department, or graduate program.

*Element 3 - Providing Support Resources.* Roles and responsibilities on the mentoring team, and even roles among the broader mentoring community, could be defined and clarified, along with recommended best practices for guiding a trainee through the development of an IDP. What additional tools and techniques can be identified to effectively manage the mentoring team? How should progress toward fulfilling the objective of the IDP be tracked? How can professional development, research, and educational goals best be aligned throughout the training process?

## Pilot 3: Building a Successful Mentoring Team

**Contributors:** Shandrea Stallworth, Valerie Fraser, Natalie Henkhaus, Katie Murphy, Andre Naranjo

This program would assist undergraduate and graduate students, postdocs, and young faculty members in developing meaningful and useful mentor/mentee relationships and teams in academia and industry. The goal of the program is to build relationships among students, faculty, and industry partners to better assist students in succeeding in their chosen career field.

Mentoring teams would consist of one of each: new faculty members, industry representatives, postdocs, graduate students, and undergraduate students. The goal is to allow industry representatives and new faculty members to grow young scientists into the careers that they see themselves in and to ensure that the scientists are able to perform in their industry. Mentoring teams allow for “no stone to be left unturned” by providing significant interactions to students throughout their academic and professional career. Mentoring teams, outside of the students’ advisor(s), give students the opportunity to build relationships with individuals who have their best interest at heart and want to see them succeed in their chosen field, not the one the adviser sees them in. Mentoring resources would be made available for both partners **(37, 38)**.

To achieve these teams, Plantae would serve as a resource to provide a large pool of potential mentors and mentees **(39)**. Plantae could be used to match individuals based on specific interests and goals. To increase participation from industry representatives, we propose advertisement for companies participating in the pilot program in return for access to the mentors and mentees. Program participants would receive financial assistance to attend PSRN conferences, private access to sponsored events such as coffee breaks and mini-receptions, and workshop-based training to assist with professional development.

To measure the effectiveness of the program, evaluations from mentors and mentees would be completed two to four times a year. Program participants would also be provided materials to assist them throughout the program. The pilot would run for two to three years with a six month to one year ramp-up to ensure thorough program development. A small feedback session during the ramp up would allow for input from faculty, industry, and students to understand what needs should be met during the program. Based on feedback, a strong mentoring team program can be developed that benefits all participants.

## Pilot 4: Developing and Credentialing Training Modules

**Contributors:** Report writing committee

Universities in their legacy operational models are neither accustomed, incentivized, nor particularly well set up to support experiences acquired outside of their physical location(s) and formal partnerships. But the needs and desires of the consumer (both the student and future employers) are changing, and universities are motivated to evolve to maintain their attractiveness **(40)**. The following mechanisms would test and explore the implementation of modular learning experiences:

*Element 1 - Complementary experience grants.* Funding agencies could offer short-term fellowships for students to undertake research or learning activities distinct from their main research project. In the private sector, support might be combined from the fellowship (travel/living) and the host (stipend). Pilot 10 would create regional hubs to connect trainees and employers for this purpose.

*Element 2 - Credentialing.* Credentialing models for modular learning could be piloted, or existing ones could be enhanced or optimized for plant science. Existing organizations, such as scientific societies, might choose to set up credentialing mechanisms, or community-based models could be supported on a competitive basis. Also, degree-granting institutions could experiment with offering reciprocal credit with other institutions through student exchanges or could create arrangements with e-learning providers to share revenues in return for granting credits. Alternatively, a consortium of degree-granting institutions could offer credit via open badging, an emerging system for verifying skills and achievements **(41)**. Integrating new and existing credentialing models will be critical. One incentive will be that prospective trainees will tend to seek institutions that are willing to credit their prior training and also encourage external training as part of their own programs. For transparency, training opportunities should be accompanied by information regarding stakeholder acceptance of credit to be obtained. Engaging employers from all sectors as credentialing systems are being developed will also be vital to ensure that the systems are relevant and useful to all.

*Element 3 - Warehousing.* A repository of accredited modules goes hand in hand with credentialing. Aggregating and organizing a meaningfully delimited set of learning opportunities is an exercise in information science. For example, a collection of thousands of MOOCs without a sophisticated search engine might thwart all but the most determined trainee. Therefore, plant (and life) science will require its own databases housing the fairly eclectic collection of opportunities that trainees may be seeking. Exploratory concepts for implementing such a resource could be funded on a pilot basis.

## Pilot 5: Industry and Academia Conference for Students

**Contributors:** Emma Frawley

**Synopsis**

The final day of the September 2017 workshop largely centered on developing tangible objectives and strategies for improving the training of plant science students. Workshop attendees emphasized pressure about the polarity of plant science career paths (i.e., “academia” vs. “industry”) and further noted a lack of awareness and opportunity to learn about industry employment—especially for students who do not attend school in research hubs like Silicon Valley, the Durham-Raleigh Research Triangle, or St. Louis.

As a result, we propose the development of a two- to three- day conference with two main objectives: (1) foster an environment to understand and improve the relationship between academia and industry, and (2) facilitate trainee (undergraduate, graduate, and postdoctoral) networking with plant-science related industries. The conference would follow several organizational structures to ensure its success: its attendees must be at the postgraduate (e.g., not faculty/supervisory) level, the cost of attending the conference for trainees would be covered by tiered entrance fees for industries or student travel grants, and there would be an application process for trainees, industry, and academia representatives, requiring a commitment to the conference objectives of educating students about diverse career opportunities, the skill sets needed to thrive in an industry environment, and a future-forward perspective on training the next-generation workforce.

We envision the conference agenda as a mix of lectures from industry representatives, open-forum panels with academic and industry delegates, trainee-run lightning talks, and low-stakes mixers to make informal connections with potential employers and mentors. Importantly, the lecture series functions not as a “business pitch” opportunity, but rather as a *resource* for trainees to learn about necessary competencies and future expectations for the company in relation to student development. Furthermore, open-forum panels serve as a space for discourse around common contentions and best practices between academia and industry, such as intellectual property, transparency, public perception, research collaborations, and the role of education in developing employable students. We suggest that trainees devise and submit questions for the panels prior to the conference for anonymity. Lastly, to include trainee participation in the conference, trainees could deliver 3- to 5- minute lightning talks to share their own ideas on how to improve relationships and communication between industry and academia, among other topics. A selection process including abstract submission would be required to participate in the lightning talks.

A variety of industry and corporate representatives would be selected to participate in the conference—from corporate incubators, to small businesses and local startups, to well-established conglomerates. This presents smaller corporations with an opportunity to publicize and brand their science and incentivizes larger corporations to participate in a form of public and community outreach. Ideally, the conference would have a mix of representatives from agriculture, biotechnology, science communication and policy, and more.

This conference is unique in that at its core, it revolves around student development and engagement, all the while attempting to cultivate cohesion between academic and corporate visions for the future. By providing trainees with the opportunity to network and learn about diverse career paths, we can open the door for them to hone their own skills and passions through higher education. Adequately educated and mentored students will succeed in a variety of career environments, including industry, a result that ultimately benefits both their academic institutions and future employers.

## Pilot 6: Science Communication Training

**Contributors:** Nicole Forrester, Nat Graham, Chris Barbey

**Motivation**

Communication skills are essential for successful careers in science, yet students and researchers have limited opportunities to acquire these skills during their academic training. Specifically, scientists must be able to effectively discuss research methods and findings with other scientists, politicians, stakeholders, industry employees, and the public to contribute to solving global issues. Although training in science communication can be conducted within institutions or at workshops and conferences, young scientists often have limited institutional resources, funding, and time to receive this training. To address this gap in training, we propose a series of free online videos focused on communication skills that will be available to K–12, undergraduate and graduate students, and postdoctoral scientists. These videos will provide fundamental training in science communication as well as enable scientists with skills geared toward a variety of science careers beyond academic research.

**Program Goal**

Enable young scientists to communicate the value and impact of scientific research with the public through free online training programs.

**Design**

**Science Communication 101**

We will create a series of 5- to 10- minute videos to aid students in developing a robust, professional skill set in science communication. Each video will focus on a key aspect of communication: for example, written, visual, and oral communication skills. These videos will be designed and created by experts within the field of communication with input from individuals with backgrounds in scientific research. The science communication series will be shared at plantae.org/AmpliComm.

**Assignment Format and Program Assessment**

Each video will be accompanied by an optional assignment specific to the skill highlighted in the video. For instance, in the written communications video, students can write a short description of their research for a public audience. These assignments will be uploaded to a website where they will be peer-reviewed/edited by other students, instructors, and teaching assistants. These assignments will not only help students implement the skills they learn from the videos, but can also be used to assess the effectiveness of the training program.

**Credentials**

Students that choose to complete all assignments for the video series will receive a certificate in Science Communication from the American Society of Plant Biologists and the National Science Foundation. Once a student receives this credential, they will be permitted to enroll in the *Specialized Communication Skills* video series. Additionally, they will be able to work as a teaching assistant where they can provide feedback to new students and catalyze discussions about science communication.

**Specialized Communication Skills for Diverse Science Careers**

To prepare young scientists for diverse careers beyond academic research, specialized programs catered toward distinct career paths (e.g., industry, journalism, teaching, and outreach) will be created. These videos will provide more detailed information and assignments specific to these career paths, as they will be made by established professionals within those fields. These programs will be recognized by additional credentials and enable young scientists to be better prepared for diverse careers in plant science.

**Online Format**

To ensure students have equal access to training opportunities in science communication, all videos will be freely available through an online platform, which will be used for uploading assignments and discussion forums. Additional resources relevant to science communication as well as career opportunities (e.g., internships, fellowships) will also be available to students.

**Update on Pilot 6.** The program described here is currently under development by a team of early career trainees and will be launched on Plantae. See plantae.org/AmpliComm for more information.

## Pilot 7: Diversity Workshop to Increase Participation of Underrepresented Groups in the Plant Sciences

**Contributors:** Andrea Carter, Chelsea Pretz, Ashleigh Farmer, Nathan Vega

Top of Form

Bottom of Form

The purpose of this workshop is to bring together representatives from industry and the academic community, including students and administrators involved in student diversity programming, to discuss how to increase involvement of underrepresented groups in plant science. It is hoped to spark a rich dialogue rooted in the different perspectives, experiences, and expectations of participants. Diversity is desired by industry and universities alike; however, the means by which we create a more diverse student body and workforce often remains more abstract than acted upon.

This workshop will be novel in its inclusiveness. Faculty members, advisers, and administrators leading or managing student diversity programming would be invited to attend along with one to two plant science students from their university that are currently involved in a diversity program (e.g., scholarship, fellowship, or mentorship program). Ideally the participating administrators would be specialized in STEM field student recruitment and retention. The administrators of diversity programs will have the opportunity to share what has worked at their institutions as well as learn from the successes and challenges faced at other schools. Lessons learned from other administrators will help inform better programming back at their own campuses as will feedback from industry representatives.

Participating students would be either undergraduate or graduate level, and self-identified as part of an underrepresented group including the following: ethnic minorities, first-generation college students, low-income, community college transfer, older/nontraditional, and different gender/sexual orientations. Students will have the opportunity to share their experience as an underrepresented plant science scholar. They will be asked to share what has specifically helped and hindered their academic career and how programming could be improved to ensure their success. The use of students’ firsthand experience will be key to improving diversity efforts at the university and industry level.

Invited industry representatives will be members of human resource departments or those some way involved in diversity initiatives at plant science–related companies. By attending this workshop, they will be able to express their expectations and desires for future employees as well as learn what they can do to create and attract a more diverse workforce.  In addition, they too will be able to learn from one another by sharing diversity efforts that have and have not worked at their respective companies.

A first of its kind, this workshop will be used to produce actionable recommendations that universities and industry can implement to ensure diversity becomes a realized aspect of the plant science field.

**Update on Pilot 7.** The program described here has been adapted into a PSRN workshop proposal, which will take place in collaboration with HHMI in early 2019. The workshop will use scenario-based thinking to discover novel paths for increasing diversity and inclusion.

## Pilot 8: Mays: Navigating and Networking Your Career in Plant Science

**Contributors:** Megan Kelly, Megan Sylvia, Crispin Taylor

**Preamble**

A lack of readily available information regarding career pathways in plant science represents both a barrier to entry and an ongoing frustration for trainees. We propose a multimedia approach to address this problem. The solution—dubbed “Mays” to represent both a connection to the crop plant species *Zea* *mays* and the fact that one may pursue multiple opportunities and pathways in a plant science career (“maze”)—combines an app, Plantae profiles, and online information and videos, along with a gaming tool to assist exploration. Mays will explain different careers and career trajectories to students and trainees, and it will facilitate direct (online) connections among early career scientists and those who’ve already taken a few steps along a particular career pathway.

**Details**

- Pathways Map: A central feature is a road map that includes stops along the way (e.g., degrees), as well as destinations (specific jobs/careers). Thickness of the road indicates the numbers of people who typically traverse that route.
- Individuals navigating Mays would tag their own pathways and stops, in much the same way that users of mapping/driving apps crowdsource information of utility to all.
- Information will be available both as general descriptions of each position or topic and as specific profiles of scientists available for networking.
- Specific information tailored to cohorts—e.g., if an undergrad thinks they want to go to grad school, they need to know that they should get some research experience.
- All information will be tagged and structured to maximize discoverability and utility in terms of navigating and exploring distinct pathways. Core information for profiles might include qualifications (and dates earned), years in specific position, salary ranges, and topical keywords.
- Information can be sliced and diced in various ways—by position, qualification, and employer type, for instance, as well as by topical areas (e.g., “food security,” “discovery research,” “breeding technologies,” “ethnobotany”)
- Mays profiles, hosted on Plantae, would include a “being a scientist” component—individuals telling their stories through written word or videos.
- Contact information would be included; this is to facilitate both peer-to-peer networking and networking among trainees and those further along their career pathway. These conversations could take place on Plantae, whether in private or in public. Participation would be incentivized in various ways, including discounts, badges, and networking opportunities. Employers would be encouraged to have employees participate in the website/app.
- Gamify with quiz aimed at keyword matching between a student/trainee’s interests and people whose profile is on the site.

**Diversity**

- Profiles of “scientist of the week” would allow us to highlight diversity (ethnic; gender; but also career type—e.g., someone working at a small/startup company)

**Paying for It**

- Mays would be monetized through job ads, institutional subscriptions (for large institutions), and individual toll access (for those at small institutions). We would adopt a “freemium” model, in which a portion of the content is freely available, lowering barriers to entry and providing enough useful information for, say, high school students. Higher-order capabilities, including networking and conversations with mentors and guides, would require payment.

**Metrics**

- We would rely on a star rating system to assess users’ appreciation for particular functions and/or pieces of content in the program.
- We would use focus groups to directly assess the utility of the site. An initial assessment would gauge understanding of career pathways in plant science and the extent of a trainee’s network. Following a few weeks on the site, we’d assess again, using a control group without access to the site.
- More mundane online usage statistics would also be tracked—IP addresses, page views, etc.

**Getting Started**

- Initial technical development will be via an app design competition, driven by detailed technical specifications generated in collaboration with ASPB digital strategy staff.
- Mays will grow over time, both helping to seed and benefiting from the growth of the Plantae network. We would test and launch with a minimum viable product that contains a few dozen key/well-traveled pathways, but would aim to rapidly increase the breadth and depth of information included in the program through outreach and engagement of plant scientists in various professions and workplaces.

## Pilot 9: Pop-up Leadership Academy

**Contributor:** Hallie Thompson

Throughout the September 2017 workshop, attendees discussed the necessity of soft skills, or non-mainstream skills for graduate-level scientific training. Furthermore, modes of education and professional development outside of the traditional scope of graduate and postdoctoral education were underscored to avoid reliance on institutional changes, which are often incremental.

We propose a pop-up leadership academy, bringing together the concepts of training scientists in nontraditional skill sets via venues that do not rely on classic education through universities or laboratories. This academy has three main objectives: (1) train a subset of conference attendees on leadership best practices; (2) instill a culture of continued learning in leadership and management and counter harmful barriers to professional development; and (3) develop a sustainable model for training continuation via volunteer curation.

The academy will fulfill initial criteria designed to achieve the above objectives, but will remain flexible enough to change upon information collection. More specifically, this will be a plant scientist leadership module designed to travel from conference to conference and across the country. The module will need to be appealing to encourage a variety of involved parties. This will be achieved through an active training design placed within conference concurrent sessions and will provide a change of energy for attendees, engaging them for the academy and preparing them for the next conference lectures. The program will be adaptable enough to apply to a variety of plant science–related conferences. Programming will focus on students but will be open to students through faculty and structured to challenge limiting assumptions around leadership. A point of focus will be the value of reflective practices in a research setting, also an important leadership habit among other professionals. Team science will be another core tenet of the training, as the necessity of collaboration grows in plant science.

Certification will be available upon course completion. To encourage volunteer involvement two levels will be included: (1) single course completion and (2) teacher in training certification. Those that complete the teacher in training certification are eligible to volunteer as staff at a future pop-up leadership academy and will be eligible to train independently in the future pending availability. Volunteers and coordinators of the pop-up will also be eligible for complimentary conference registration.

Preparing Tomorrow’s Leaders for Science is a student- and postdoc- oriented leadership program in existence at the University of Missouri in Columbia. It is a year-long program and focuses on a broad spectrum of leadership skills and the applications of these to science. The pop-up academy will utilize the expertise available through the development of this program and others across the country. However, a plant scientist leadership academy traveling to and from conferences is unique in that it makes this brand of training available to students from a larger number of institutions and, in turn, may inspire local courses or programs. Ultimately this will result in student ownership of training and an increase in high-utility skills among plant scientists, no matter their future career path.

## Pilot 10: Creating Active Participants Out of Trainees

**Contributors:** Andrew Nelson, Navadeep Boruah, Bethany Huot, Irene Liao

**Background**

Transition years, defined as the year following each of the different stages of academic training (high school → undergraduate → graduate school → postdoc), are a time of enormous uncertainty for most aspiring scientists. Many promising young scientists lack a clear vision of their available career/education options and thus proceed along the traditional training pipeline by default. This is a problem for several reasons, including fit (many may be happier in non-academic positions), supply and demand (there are not enough academic research positions available), and societal need. Also, while this pipeline funnels trainees toward academic research positions, it provides little training for the actual tasks performed in such positions. In spite of the fact that five out of six will not obtain an academic position, career paths outside of this sector are typically not identified in a purposeful manner. To break down this unsustainable and wasteful training regime, a program that effectively exposes trainees to the diverse array of postgraduate careers available to them as plant scientists is needed. Ideally, this training program would be dynamic and driven primarily by the trainee’s needs and interests. If successful, this program would match strong candidates with all possible career paths, thereby reducing the oversaturation of the academic job market and better supplying the demand for scientists with versatile and diverse skill sets. As the path through education becomes more successful, and actively chosen careers in diverse job sectors become the norm, we anticipate an increase in the number and diversity of students choosing to pursue higher education in plant science.

**Proposal Specifics**

As a pilot program, this proposal encourages the funding of one or two regional training hubs consisting of academic, industry, and affiliated plant science groups (e.g., science policy experts, K–12 science education programs). These hubs would be the site of transition-year training programs, targeting competitive candidates who are within a year of graduating high school or completing a bachelor’s, master’s, or Ph.D. program as potential recruits. Trainees would spend a year in this program, sampling different research/affiliated groups and acquiring the transferable skills necessary to take ownership over their future.

**Exposure to Diverse Research Groups**

As part of the program, trainees would rotate through a minimum of three to four groups in various job sectors. Trainees could negotiate with mentors/advisers on length and goals of each particular rotation. Where appropriate, rotations could occur concurrently, allowing for a more organic training process. The focus of rotations would be less on completing a particular project and more on sampling different research environments/cultures within the various partnering sectors.

**Mentoring**

For mentorship through the training program, each trainee would be assigned three advisers from academic, industry, and affiliated groups based on their career interests, who would provide advice and valuable insights into each of these sectors. In addition, this proposal would pay for the hiring of a career placement adviser who would assist trainees in examining future job or educational prospects. Finally, trainees within the program would be paired with other trainees further along in their professional development to foster mentoring skills and encourage exchange of experiential knowledge.

**Soft Skill Development**

Alongside rotations, trainees would develop soft skills through a series of workshops and public outreach programs. Soft skills development workshops would contain content preselected by the training program, with topics centered around making and giving presentations, all aspects of manuscript preparation and publication, tips for interviewing and putting together applications, and considerations for establishing and maintaining collaborations. Trainees could then choose additional content specific to their interests (e.g., grant writing, composing budgets, and personnel management). These workshops would be taught by stakeholders within the program or by consultants. Soft skill development through public outreach programs would occur by partnering with local organizations to bring “digestible” science to the public, such as to K–12 classrooms, Rotary and Lion’s Club meetings, and assisted living homes. With assistance from the career placement adviser, trainees would develop appropriate activities to engage with the public about the importance of plant science. Additionally, trainees will be involved in the process of putting together such an activity, thereby building leadership, communication, networking, and organizational skills.

**Technical Skill Development**

In addition to focusing on soft skills, short (one to two week) courses would discuss technical skills being utilized by the various partners in the program. Technical skill development would begin with introductory “courses” in the various skill categories being offered (e.g., genetics, bioinformatics, biochemistry, statistics, education, policy), focusing on how the different approaches can address applied or basic research questions. From there, trainees would be able to select from different modules based on preference, without prerequisite, and in a nonlinear fashion. Skill set courses could be taught by any member of the participating partners’ groups. The prerequisite for teaching a skill set would be proof of that skill (published or acknowledged by peers). A course rating system would inform future trainees.

**Assessment**

The success of this training program will be assessed through evaluations and reflections. Trainees will be asked to evaluate their perceived prospects prior to participating in the program, and then on a yearly basis for five years after leaving the program. Similar to the peer review process, trainees will give both private and public reviews for workshops, modular training, and rotations following completion. These evaluations and feedback will also be used to constantly improve the program in order to provide the tools and experiences that the participants seek and require as the times change. Participants’ professional positions will also be tracked to see how well this program helped participants reach their career goal.

**Sustainability**

This program would initially form as a collaboration among industry, academia, and other interested partners. During the first year, NSF or other funding agents would absorb 50% of the program cost (stipends, salary for career placement adviser, workshops, etc.), with the other 50% split evenly among partners. Over the lifetime of the program, support from funding bodies would diminish (e.g. 5–10% per year), with increasing support from participating partners. Academic partners would be offered supplemental support on existing NSF grants to cover costs. Although unnecessary, endowments could also be pursued to minimize long-term costs to the program. As an additional incentive, participating partners would have first chance at recruiting from a well- and broadly trained group of scientists.

**Conclusion**

The number and diversity of trainees choosing to pursue higher education in plant science may be restricted by a lack of training tailored toward specific, visible career outcomes. Providing access to knowledge, resources, and experiences that facilitates trainee ownership and strategic career management will help trainees develop the transferable skill sets and confidence needed to actively choose their future career. We also expect this program to reduce the length of time it takes for trainees to obtain their desired career by maximizing fit through tailored training and helping establish a network within the job sector of their interest. If these outcomes are realized, we anticipate an increase in the number and diversity of students drawn to higher education within plant science.
